# A CMOS Smart Temperature and Humidity Sensor with Combined Readout

**DOI:** 10.3390/s140917192

**Published:** 2014-09-16

**Authors:** Clemens Eder, Virgilio Valente, Nick Donaldson, Andreas Demosthenous

**Affiliations:** 1 Department of Electronic and Electrical Engineering, University College London, Torrington Place, London WC1E 7JE, UK; E-Mails: c.eder@ucl.ac.uk (C.E.); v.valente@ucl.ac.uk (V.V.); 2 Department of Medical Physics and Bioengineering, University College London, Malet Place, WC1E 6BT, UK; E-Mail: n.donaldson@ucl.ac.uk

**Keywords:** capacitive sensor, humidity measurement, microelectronic implants, oscillator, on-chip sensor, temperature sensor

## Abstract

A fully-integrated complementary metal-oxide semiconductor (CMOS) sensor for combined temperature and humidity measurements is presented. The main purpose of the device is to monitor the hermeticity of micro-packages for implanted integrated circuits and to ensure their safe operation by monitoring the operating temperature and humidity on-chip. The smart sensor has two modes of operation, in which either the temperature or humidity is converted into a digital code representing a frequency ratio between two oscillators. This ratio is determined by the ratios of the timing capacitances and bias currents in both oscillators. The reference oscillator is biased by a current whose temperature dependency is complementary to the proportional to absolute temperature (PTAT) current. For the temperature measurement, this results in an exceptional normalized sensitivity of about 0.77%/°C at the accepted expense of reduced linearity. The humidity sensor is a capacitor, whose value varies linearly with relative humidity (RH) with a normalized sensitivity of 0.055%/% RH. For comparison, two versions of the humidity sensor with an area of either 0.2 mm^2^ or 1.2 mm^2^ were fabricated in a commercial 0.18 μm CMOS process. The on-chip readout electronics operate from a 5 V power supply and consume a current of approximately 85 μA.

## 1. Introduction

Temperature and humidity sensors are widely used in many measurement and control applications, including process control, meteorology, agriculture, battery-powered systems and medical equipment [1–8]. Temperature is a major concern in active implanted medical devices, especially in situations where neural stimulators are located in close proximity to the neural tissue [9]. To protect patients from harm due to the heat dissipated from implantable stimulators, the ISO 14708-3 [10] requires that no outer surface of an implantable part be greater than 2 °C above the normal surrounding body temperature, either in normal operation or single-fault condition. Any higher temperature rises can only be justified if the manufacturer convincingly demonstrates its safety for a particular application [10]. An on-chip temperature sensor calibrated for a small temperature range (e.g., 35 °C to 40 °C), but which is very sensitive to small changes (e.g., 0.1 °C) will ensure the safe operation of a stimulator located on the same chip. The requirements for sensors used for on-chip thermal management of non-implantable processors are different due to their allowable wider temperature range and the stability of the supply voltage [11,12]. While it has been demonstrated that a high level of absolute accuracy can be achieved with a complementary metal-oxide semiconductor (CMOS) smart temperature sensor [13], relative changes (from the initial value on implantation) are more important for implant monitoring, and they require a high level of sensitivity.

Moisture is another major concern for electronic devices operated in humid environments or implanted in the body. Moisture that has penetrated the device package eventually causes condensation on the active area of the integrated circuit, leading to corrosion, performance deterioration and device failure. The most direct way to check that the micro-package [14] is functional and remains dry is to measure its internal relative humidity (RH). A humidity sensor using a 0.6 μm CMOS process with on-chip readout electronics is reported in [15]. An interdigitated capacitor that is covered by an inorganic passivation layer followed by a polyimide overcoat forms the moisture sensitive sensor. It does not require any post-processing steps. However, the area required for the sensor itself is relatively large (4 mm^2^ in [15]). It is desirable to provide a small sensor that can be added, for example, to micro-packaged implantable stimulator chips [9,16] without a large increase in total area.

This paper presents a combined temperature and RH sensor with a common readout, which simplifies the design and significantly reduces the chip area needed. The readout is based on ratiometric counter values in which a measurement counter is driven by a relaxation oscillator. Its frequency depends on either a temperature-dependent current charging a constant capacitor or on a constant current charging a humidity-dependent capacitor. By associating the reference counter to a current with opposite temperature coefficients, the temperature sensitivity can be increased (as will be shown). The combination of small area, supply voltage independence, high sensitivity to both temperature and humidity changes, as well as the ease of readout makes the design concept very attractive for active implantable epidural electrodes incorporating several stimulator chips [16]. For monitoring purposes in active implantable microsystems, it is the relative changes in temperature and humidity (and not their absolute values) that are important in order to trigger an alarm.

The paper is organized as follows. Section 2 describes the concept overview, and Section 3 provides a sensitivity analysis for both humidity and temperature. Section 4 presents details of the circuit design. The measured results follow in Section 5. Finally, the discussion and concluding remarks are presented in Sections 6 and 7.

## 2. Architectural Overview

**Figure 1.**
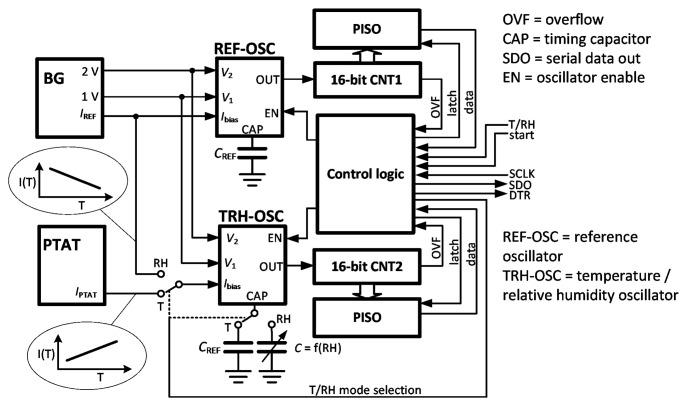
Architecture of the combined temperature and humidity sensor. Depending on the measurement mode, one of the counters (CNT1 or CNT2) defines the reference period for the other counter. BG, bandgap circuit; REF-OSC, reference oscillator; T/RH, temperature/relative humidity; PTAT, proportional to absolute temperature; PISO, parallel-in/serial-out; DTR, data ready; SCLK, serial clock.

Figure 1 shows the architecture of the combined temperature and humidity sensor. The readout is based on the ratiometric frequency measurement of two relaxation oscillators. Two modes of operation are selectable by the T/RH control signal, either the temperature mode (TMOD) or the relative humidity mode (RHMOD). The operation of the reference oscillator (REF-OSC) is the same in both modes. A bandgap circuit (BG) generates temperature-independent voltage levels of 1 V and 2 V, which are used to set the thresholds of the window comparators in the oscillator. REF-OSC is always connected to the same reference capacitor (*C*_REF_) and supplied by bias current *I*_REF_, biasing its internal current source/sink. This current is generated in BG and has a negative temperature coefficient; therefore, the frequency of REF-OSC decreases with an increase in temperature.

In the TMOD of operation, REF-OSC and one 16-bit counter (CNT1) generate a reference time interval. Any temperature increase leads to an increase in the proportional to absolute temperature (PTAT) current, increasing the frequency of the readout oscillator (TRH-OSC) and the rate at which the readout counter CNT2 counts. When CNT1 overflows, the instantaneous value of CNT2 is sampled. Note that the frequencies of REF-OSC and TRH-OSC are moving in opposite direction with increasing temperature, which improves the sensitivity of the temperature measurement. The consequences for linearity will be elaborated in Section 3.

In the RHMOD of operation, the frequency of TRH-OSC decreases with increasing capacitance, which is directly proportional to RH (*i.e.*, *C* = f(RH)). In this mode, the roles of CNT1 and CNT2 are therefore interchanged, where CNT2 defines the measurement interval and CNT1 is engaged for readout. Note that TRH-OSC is biased by the same current as REF-OSC; thus, to a first order approximation, the temperature dependence of the current is cancelled. The frequency measurement is only affected by changes of the sensor capacitance, which ideally depends on humidity only. The following analysis examines the performance of both modes of operation in terms of sensitivity and linearity.

## 3. Sensitivity Analysis

**Figure 2.**
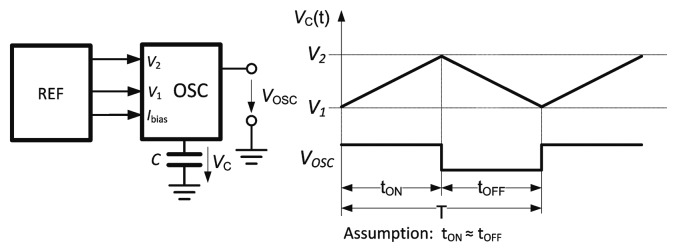
Triangular waveform on the capacitor and output waveform as a function of time. The duty cycle is assumed to be 50%.

The timing of the oscillator waveform is shown in Figure 2. During one half period (*t*_ON_), the capacitor *C* is charged from the comparator reference voltage level *V*_1_ to *V*_2_ and:

(1) $$\Delta V=(V_{2}-V_{1})=\frac{1}{C}\int_{\tau =t}^{\tau =t+t_{\text{ON}}}I(t)d\tau$$

For a bias current *I*(*t*) = *I* and a 50% duty cycle:

(2) $$\Delta V=\frac{1}{C}\cdot I\cdot t_{\text{ON}}\Rightarrow f_{\text{OSC}}=\frac{I}{2C(V_{2}-V_{1})}$$

where *f*_OSC_ is the oscillator frequency. The frequency is directly proportional to current and inversely proportional to capacitance. Consequently, the roles of the reference and measurement counters must be interchanged when switching from one mode to another. CNT1 is the reference counter for TMOD, while CNT2 is for RHMOD. The two modes of operation are therefore treated separately below.

### 3.1. Humidity Sensor Mode (RHMOD)

The measurement interval, *T*_RH_, is determined by the TRH-OSC frequency, *f*_RH_, and the bit size of the overflow counter, CNT2. For a 16-bit counter, the measurement interval is:

(3) $$T_{\text{RH}}=\frac{N_{\text{CNT2}}}{f_{\text{RH}}}=\frac{2^{16}}{f_{\text{RH}}}$$

where *N*_CNT2_ is the counter value of CNT2. During this interval, the reference counter counts up to a value *N*_REF_, which depends both on the frequency of the measured signal and the measurement interval. Hence,

(4) $$N_{\text{CNT1}}=f_{\text{REF}}\cdot T_{\text{RH}}=\frac{f_{\text{REF}}}{f_{\text{RH}}}\cdot 2^{16}\pm 0.5$$

where *f*_REF_ is the REF-OSC frequency. The uncertainty of half a count, *i.e.*, the quantization error, is taken into account. Equation (4) shows that the reference counter value is a measurement of the frequency ratio of the oscillators. As the frequencies are dependent on both current and capacitance (see Equation (2)), the frequency ratio may be expressed as the ratiometric measurement of either currents or capacitances:

(5) $$N_{\text{CNT1}}=\frac{f_{\text{REF}}}{f_{\text{RH}}}\cdot 2^{16}\pm 0.5=\frac{I_{\text{REF}}(T)}{C_{\text{REF}}(T)}\cdot \frac{C_{\text{RH}}(T,\text{RH})}{I_{\text{REF}}(T)}\cdot 2^{16}\pm 0.5$$

where *C*_REF_ is the reference capacitor, *C*_RH_ is the humidity sensitive capacitor, *I*_REF_ is the REF-OSC current and *T* is the temperature. It is assumed that neither the capacitance of the reference capacitor nor the amplitude of the current is affected by RH. For equal bias currents, the sampled counter value *N*_CNT1_ is therefore only dependent on the capacitive ratio *C*_RH_/*C*_REF_. Two cases are considered.

#### 3.1.1. Humidity Dependency

Equation (5) is independent of the current ratio and only dependent on the capacitance ratio, that is:

(6) $$N_{\text{CNT1}}=\frac{f_{\text{REF}}}{f_{\text{RH}}}\cdot 2^{16}\pm 0.5=\frac{C_{\text{RH}}(\text{RH})}{C_{\text{REF}}}\cdot 2^{16}\pm 0.5$$

Since *C*_REF_ can be considered constant and does not depend on RH, the change in counter value with humidity is:

(7) $$\frac{\partial N_{\text{CNT1}}}{\partial \text{RH}}=\frac{1}{C_{\text{REF}}}\cdot \frac{\partial C_{\text{RH}}(\text{RH})}{\partial \text{RH}}\cdot 2^{16}=\frac{C_{\text{RH}}(\text{RH})}{C_{\text{REF}}}\cdot \text{NSRH}\cdot 2^{16}$$

where NSRH is the normalized sensitivity to RH, which is of the order of 0.073%/% RH [15]. For equal capacitances, the counter change is of the order of 730 ppm/RH·2^16^ ≈ 48, and the least significant bit (LSB) resolution is therefore 1/48 ≈ 0.02% RH.

#### 3.1.2. Temperature Dependency

Here, Equation (6) is modified to take the temperature dependency of the reference capacitance into account:

(8) $$N_{\text{CNT1}}=\frac{f_{\text{REF}}}{f_{\text{RH}}}\cdot 2^{16}\pm 0.5=\frac{C_{\text{RH}}(T)}{C_{\text{REF}}(T)}\cdot 2^{16}\pm 0.5$$

The change in the counter value with temperature is:

(9) $$\frac{\partial N_{\text{CNT1}}}{\partial T}=\frac{1}{{\left(C_{\text{REF}}(T)\right)}^{2}}\cdot \left(\frac{\partial C_{\text{RH}}(T)}{\partial T}C_{\text{REF}}(T)-\frac{\partial C_{\text{REF}}(T)}{\partial T}C_{\text{RH}}(T)\right)\cdot 2^{16}$$

This dependency only cancels in the ideal case when both capacitances and their temperature coefficients are equal. For any other case, the temperature coefficient of a capacitor, *TTC*, is given by:

(10) $$\text{TCC}=\frac{1}{C(T_{0})}\cdot \frac{\partial C(T)}{\partial T}$$

where *C*(*T*_0_) is the capacitance at temperature *T*_0_. A typical value of *TCC* for a poly-poly capacitor *C*_REF_ in a typical 0.18 μm CMOS process is 20 ppm/°C. Metal-dielectric-metal capacitors have higher temperature coefficients, and the simulated temperature coefficient of the top-metal humidity sensor is about 60 ppm/°C.

Equation (7) can be expressed in terms of *TTC* as:

(11) $$\frac{\partial N_{\text{CNT1}}}{\partial T}=\frac{C_{\text{RH}}(T)}{C_{\text{REF}}(T)}\left({\text{TCC}}_{\text{RH}}-{\text{TCC}}_{\text{REF}}\right)\cdot 2^{16}$$

where *TCC*_RH_ and *TCC*_REF_ are, respectively, the temperature coefficient of capacitor *C*_RH_ and *C*_REF_. For equal capacitances, the change in the counter value is about 40 ppm/°C · 2^16^ = 2.62/°C. A temperature increase of about 18 °C appears as a 1% RH increase. This temperature sensitivity is acceptable in implants where temperature variations are low.

### 3.2. Temperature Sensor Mode (TMOD)

#### 3.2.1. Temperature Dependency

The temperature sensitivity of the counter stage is first examined for a reference current *I*_REF_ with zero temperature coefficient (*i.e.*, *TCI*_REF_ = 0). The roles of measurement and reference counters are now reversed (Figure 1). Equation (5) assumes the form:

(12) $$N_{\text{CNT2}}=\frac{f_{\text{T}}}{f_{\text{REF}}}\cdot 2^{16}\pm 0.5=\frac{I_{\text{T}}(T,\text{RH})}{I_{\text{REF}}}\cdot \frac{C_{\text{REF}}(T)}{C_{\text{T}}(T)}\cdot 2^{16}\pm 0.5$$

where *f*_T_ is the TRH-OSC frequency (in TMOD), and the capacitors *C*_T_ and *C*_REF_ are of the same type with equal temperature coefficients. Thus, the derivative of the quotient (which can be derived in a similar manner to Equations (8) to (11)) is:

(13) $$\frac{\partial }{\partial T}\left(\frac{C_{\text{REF}}(T)}{C_{\text{T}}(T)}\right)=\frac{C_{\text{REF}}(T)}{C_{\text{T}}(T)}\left({\text{TCC}}_{\text{REF}}-TCC_{\text{T}}\right)=0$$

The temperature, therefore, depends only on the PTAT current (*I*_T_) multiplied by the scaling factor *C*_REF_/*C*_T_:

(14) $$\frac{\partial N_{\text{CNT2}}}{\partial T}=\frac{1}{I_{\text{REF}}}.\frac{C_{\text{REF}}}{C_{\text{T}}}(\frac{\partial I_{\text{T}}(T)}{\partial T})\cdot 2^{16}$$

where *I*_T_ is assumed independent of RH. The temperature coefficient of the current is given by [17]:

(15) $$TCI_{\text{T}}=\frac{1}{I_{\text{T}}}\cdot \frac{\partial I_{\text{T}}}{\partial T}=\frac{1}{V_{\text{EB}12}}\cdot \frac{\partial V_{\text{EB}12}}{\partial T}-\frac{1}{R}\cdot \frac{\partial R}{\partial T}=\left(\frac{1}{T}-\text{TCR}\right)$$

where *V*_EB12_ is the voltage difference between the differently biased bipolar transistors (Figure 5a) and *TCR* denotes the temperature coefficient of a resistor. Therefore, Equation (14) can be written as:

(16) $$\frac{\partial N_{\text{CNT2}}}{\partial T}=\frac{I_{\text{T}}}{I_{\text{REF}}}.\frac{C_{\text{REF}}}{C_{\text{T}}}\frac{1}{T}\left(1-T\cdot \text{TCR}\right)\cdot 2^{16}$$

The term *T*·*TCR* introduces a non-linearity. *TCR* can be very small for a poly-resistor (−40 ppm/°C), which is much smaller than the *TCI*_T_ at room temperature (about 3300 ppm/°C).

#### 3.2.2. *TCI*_REF_ < 0, Temperature Dependency

The derivative of a current ratio can be expressed in a similar manner to Equation (11) as:

(17) $$\frac{\partial }{\partial T}\left(\frac{I_{\text{T}}}{I_{\text{REF}}}\right)=\frac{I_{\text{T}}}{I_{\text{REF}}}\cdot \left(TCI_{\text{T}}-TCI_{\text{REF}}\right)=\frac{I_{\text{T}}}{I_{\text{REF}}}\cdot \left(\frac{1}{T}-TCR-TCI_{\text{REF}}\right)$$

The dependency of the relative value of CNT2 depends on temperature and is:

(18) $$\frac{\partial N_{\text{CNT}2}}{\partial T}=\frac{I_{\text{T}}}{I_{\text{REF}}}\cdot \frac{C_{\text{REF}}}{C_{\text{T}}}\cdot \frac{1}{T}\left(1-T\left(TCI_{\text{REF}}+TCR\right)\right)\cdot 2^{16}$$

**Figure 3.**
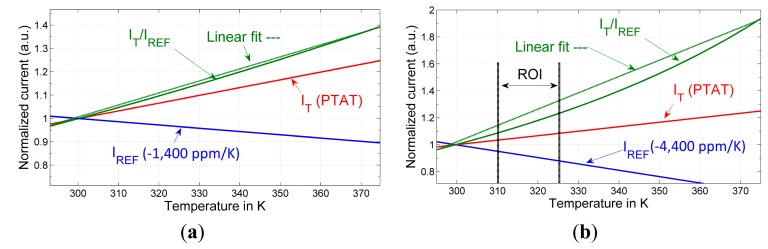
Qualitative illustration of the compromise between linearity and sensitivity of the ratio between PTAT and reference currents. (**a**) A reference current with a low negative temperature coefficient will result in increased overall sensor sensitivity while minimally compromising linearity; (**b**) Sensitivity can be further increased with a reference current of a higher negative temperature coefficient, at the cost of increased deviation from linearity.

Since the temperature coefficient of the bandgap was simulated close to zero and the temperature coefficient of the n-well resistor used in BG is *TCR* = 3000 ppm/°C, the reference current will necessarily have a negative temperature coefficient of *TCI*_REF_ = −3000 ppm/°C. Together with the chosen poly-type resistor for the PTAT current generation (−1400 ppm/°C), the sum of both coefficients is −4400 ppm/°C, which is subtracted from the 3300 ppm/°C of the PTAT current at 300 K. This presents a boost in sensitivity by a factor of 2.3 with respect to a PTAT current generator. The change in the counter value is 7700 ppm/°C · 2^16^ ≈ 505, corresponding to an LSB resolution of 1/505 ≈ 0.002 °C. However, the increase in sensitivity is at the expense of increased non-linearity. The analysis of the latter is not trivial, and a qualitative illustration of the trade-off between linearity and sensitivity is shown instead in Figure 3. As the sensor will be used for temperature monitoring in an implant where the temperature range of interest is restricted, the errors due to non-linearity are small (Figure 3).

## 4. Sensor and Circuit Design

### 4.1. Humidity Sensor

**Figure 4.**
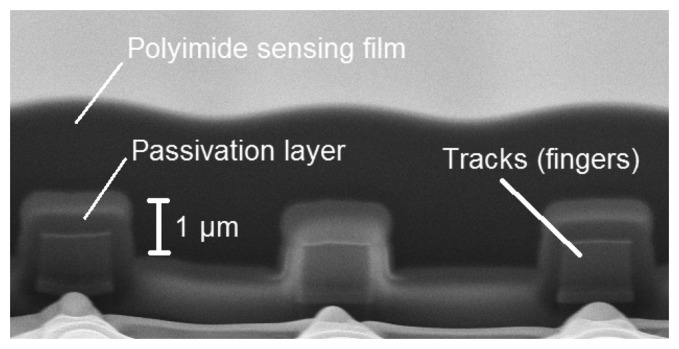
Cross-section for three of the sensing capacitor's fingers in [15] sectioned by focused ion beam.

The capacitive humidity sensor was constructed as in [15,18] and requires no post-processing steps. It is based on a capacitor consisting of an interdigitated finger structure formed by the top metal layer with its inorganic passivation coating. The structure is covered by a moisture sensitive film, which is formed by the readily available polyimide overcoat. A cross-section of the fabricated sensor in [15] is shown in Figure 4.

In the proposed design, the fingers are 3 μm wide and spaced 2.5 μm apart (the minimum width and spacing allowed by the design rules of the technology; 0.18 μm X-FAB XP018). Two types of sensor were implemented with sensing capacitors of different sizes. A 1 mm × 1 mm (15 pF simulated capacitance between fingers) and a 300 μm × 300 μm (1.1 pF simulated capacitance) were fabricated to investigate the sensitivity in these small structures. The larger sensor was accessible via pads to test its capacitance as a function of humidity. The chip also contained circuit structures enabling testing of individual blocks, such as the bandgap (BG) and PTAT reference outputs.

### 4.2. Bandgap and PTAT Designs

#### 4.2.1. Bandgap Circuit

**Figure 5.**
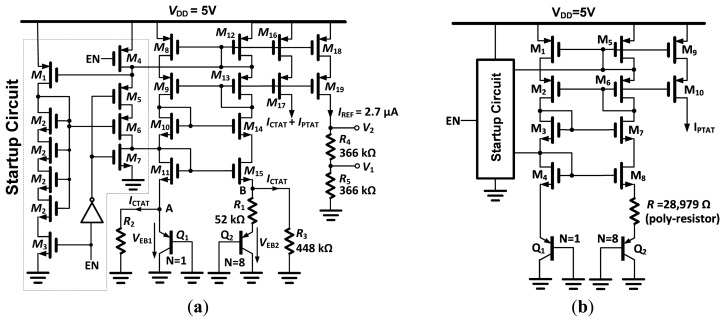
(**a**) Reference current and voltage source. All resistors are of the n-well type; (**b**) PTAT current source.

Both the temperature-independent voltage references *V*_1_ and *V*_2_, and a temperature-dependent reference current *I*_REF_ are generated (Figure 5a). The topology is based on summation of a PTAT current and a complementary-to-absolute temperature (CTAT) current, which is a commonly used design in short channel processes [17]. A self-biased cascode current source (*M*_8_–*M*_15_) is used to hold Nodes A and B at the same voltage, *V*_EB_ (base-emitter voltage) of *Q*_1_. The cascode current mirror requires about 2.3 V of compliance. The PTAT current is generated via the *V*_EB1_–*V*_EB2_ difference over *R*_1_ and added to the CTAT current generated by *V*_EB1_ over *R*_2_ and *R*_3_. The sum of the currents is mirrored to *R*_4_ and *R*_5_, which generate temperature-independent voltage drops of 1 V (*V*_1_) and 2 V (*V*_2_). The temperature dependence of *V*_1_ or *V*_2_ is a function only of the ratio of the values of *R*_2_ and *R*_1_ and the number (*N* = 8) of elements used in *Q*_2_ [17]. All resistors were selected as the n-well type, because of the positive temperature coefficient of about 3000 ppm/°C. This yields a negative *TCI*_REF_, as previously explained.

#### 4.2.2. PTAT Circuit

The PTAT current source is shown in Figure 5b, and its topology is similar to the bandgap reference (without the CTAT current). The poly-resistor has a negative temperature coefficient of −1400 ppm/K, in order to further increase the temperature sensitivity of the output current. The startup circuit is identical to the one used in the bandgap reference.

### 4.3. Relaxation Oscillator

**Figure 6.**
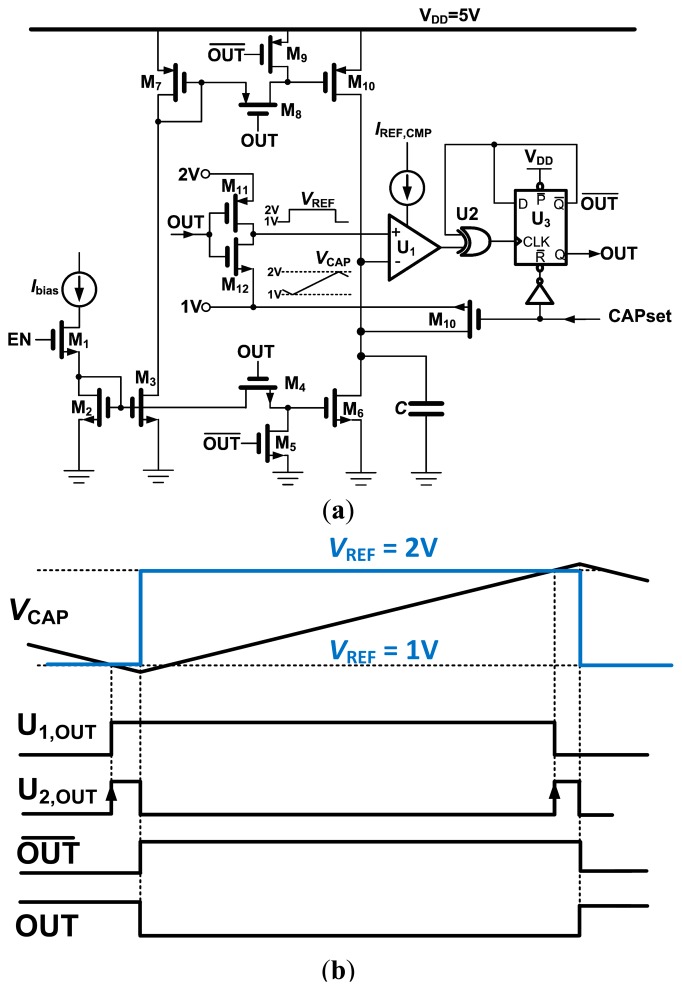
(**a**) Relaxation oscillator with a single comparator; (**b**) oscillator timing diagram (dimensions are not to scale).

The relaxation oscillator is shown in Figure 6a. Prior to the start of the oscillation, the timing capacitor *C* is connected to the high impedance node formed by the inactive output transistors of the current source (M_10_) and sink (M_6_). The capacitor set (CAPset) signal resets the output and connects the output node to the lower threshold voltage. The capacitor will be rapidly charged to 1 V, allowing even the very first charging interval *t*_ON_ (Figure 2) to be accurately defined. The oscillator starts by driving CAPset to “0” and the oscillator enable (EN) signal to “1”, enabling the bias current through the 1:1 current mirrors formed by M_2_, M_3_, M_6_ and M_7_, M_10_. Output transistors M_10_ and M_6_ are alternately switched off (by M_5_, M_9_) or connected to the current mirror through M_4_, M_8_.

This design uses a single comparator implemented as an uncompensated two-stage op-amp, saving the power consumption of a second comparator. The relaxation oscillator is based on charging and discharging the timing capacitor between two well-defined voltages. The threshold values are switched depending on whether the capacitor is being charged or discharged. This is accomplished by the XOR gate U_2_ and the D-flip flop U_3_. When the capacitor voltage reaches the lower bound, the output of the comparator is set to the positive supply rail (Figure 6b). The inverted output of U_3_ is still at 0 V, causing U_2_ to be set to “1”, which resets U_3_ and causes the output of U_2_ to change back to “0”. The duration for which the output of U_2_ stays high is mainly determined by the propagation delays of U_2_ and U_3_ and are in the ns range. The undershoot that occurs before the flip-flop toggles causes negligible timing errors (Figure 6b).

### 4.4. Control Logic

The control logic was designed in Verilog and consists of a simple state machine. A start signal first sets CAPset to high, while enabling the bandgap reference (Figure 6a). It resets the output of the D-flip flop, which connects the gate of M_10_ to M_7_. However, the current mirror M_2_–M_3_ is not enabled, thus the drain of M_7_ is at *V*_DD_ (5 V), turning off M_7_. The node at *V*_CAP_ is therefore high impedance, and the timing capacitor charges up to *V*_1_ via the switch M_10_. The time constant is defined by the capacitance and the output resistance of the circuit generating *V*_1_, which is R_4_‖R_5_ and is about 2.7 μs for the larger humidity capacitor. A pulse of 20 μs is used to safely pre-charge the capacitor before turning off CAPset and starting the triangular oscillation of *V*_CAP_ from the lower threshold *V*_1_. Each oscillator output is connected to a separate counter, whose frequency is recorded in a parallel-in/serial-out (PISO) register by the overflow of the counter (their roles are determined according to the T/RH mode signal in Figure 1). Once the data transfer occurs, a data ready (DTR) signal is set high to indicate the end of the measurement. The recorded value is then ready to be clocked out from the PISO by the external serial clock (SCLK) (Figure 1).

## 5. Measured Results

**Figure 7.**
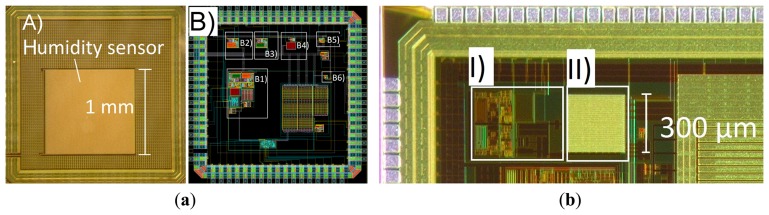
(**a**) (A) Chip microphotograph of Version 1 chip (individual blocks are not visible due to the top-metal dummy structure) and (B) layout with omitted top metal layer. Test structures: (B1) readout circuit, (B2) bandgap, (B3) PTAT, (B4) biasing stage, (B5) relaxation oscillator, (B6) comparator. (**b**) Chip microphotograph of Version 2 chip with smaller sensing capacitor: (I) readout circuit, (II) capacitive sensing element.

Two versions of prototype chips were fabricated in a 0.18-μm CMOS process (X-FAB XP018). Microphotographs of the chips are shown in Figure 7 (Version 1 and Version 2 chips). The larger 1 mm × 1 mm capacitor (humidity sensor) is visible on the top layer in Figure 7a. The readout circuit occupies an area of 350 μm × 580 μm. The different test-structures of the readout (Circuits B1 to B6 in Figure 7a) were first used to characterize the temperature and humidity sensitivity of the individual blocks. These test structures are only available in the Version 1 chip, and they represent functional blocks of the readout (which is nearly identical in both versions). The only difference in the readout in the Version 2 chip is that the control logic part is omitted. This design choice had to be made as the number of available pads was limited, and it was justified by confirming the correct operation of the control logic in the Version 1 chip.

For the sensor characterization, an MKF 240 environmental simulation chamber was used (BINDER GmbH, Tuttlingen, Germany). All measurements were performed with the test printed circuit board inside the chamber, connected by cables through access ports. Capacitance measurements were performed with a Wayne Kerr 6500B precision impedance analyser (Wayne Kerr Electronics Inc., Woburn, MA, USA). Short bursts at the output of both oscillators were sampled at 1 MHz using a NI-USB-6353 acquisition card (National Instruments, Austin, TX, USA). A frequency counter (Agilent 53131A, Santa Clara, CA, USA) was used for the direct measurement of the frequency ratio between the outputs of the two oscillators. A summary of the overall performance is given in Table 1.

### 5.1. Capacitance vs. Relative Humidity (Version 1)

**Figure 8.**
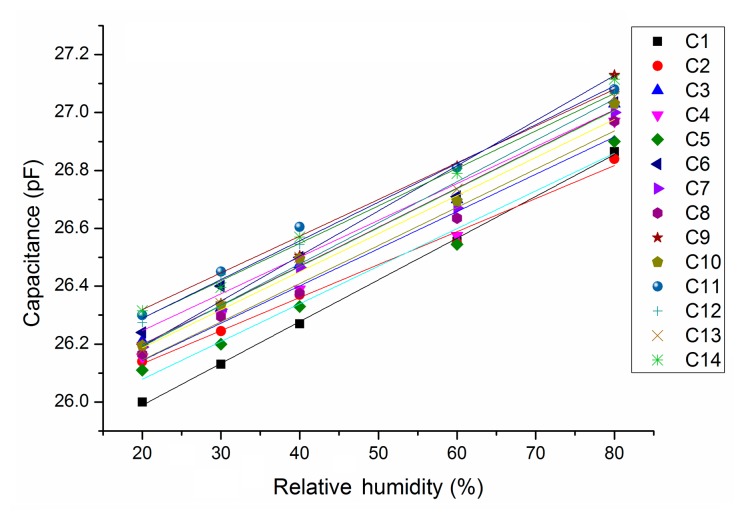
Measured capacitance *versus* relative humidity at 37 °C.

All 14 sensors of Version 1 showed a linear capacitance response as a function of humidity (Figure 8), where the coefficient of determination is greater than 0.99. The mean response is:

(19) C(pF)=0.0133·RH(%)+25.92pF

where the 99% confidence interval (CI) is (25.8, 25.99) pF for the intercept and (0.0125, 0.0141) pF/% for the slope. The normalized mean sensitivity is 0.0133/25.9 = 514 ppm/% RH at 0% RH.

### 5.2. Temperature Dependency of Reference (Version 1)

**Figure 9.**
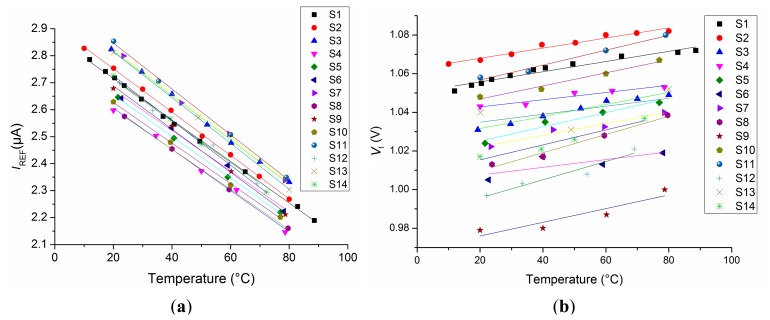
(**a**) Measured reference currents with temperature; (**b**) Measured temperature dependency of all reference voltages *V*_1_.

The reference current followed a linear trend with a negative temperature coefficient (Figure 9a):

(20) $$I_{\text{REF}}(\mu \text{A})=-0.00794\cdot T({}^{\circ}\text{C})+2.88\mu \text{A}$$

where the 99% CI is (2.81, 2.95) μA for the intercept and (−0.0082, −0.0077) μA/°C for the slope. The average sensitivity is −0.00789/2.88 μA = − 2771 ppm/°C at 0 °C. The nominal simulated response was practically identical to the measured average response, which had a slope <3000 ppm/°C. This would be the slope expected from the quoted *TCR* of the n-well resistor of 3000 ppm/°C, if the reference voltage was ideal and had a zero temperature coefficient.

The measurement of the reference voltage *V*_1_ is shown in Figure 9b. Analysis of the results showed a 99% CI for the intercept between 1 V and 1.04 V, with a mean slope of 322 μV/°C, corresponding to a temperature coefficient of 316 ppm/°C. The variation of the gradients has a 99% CI of (248, 397) μV/°C. The sensitivity of *V*_1_ to temperature variations is attributed to the spread of the characteristics of the n-well resistors.

### 5.3. Temperature Dependency of PTAT (Version 1)

**Figure 10.**
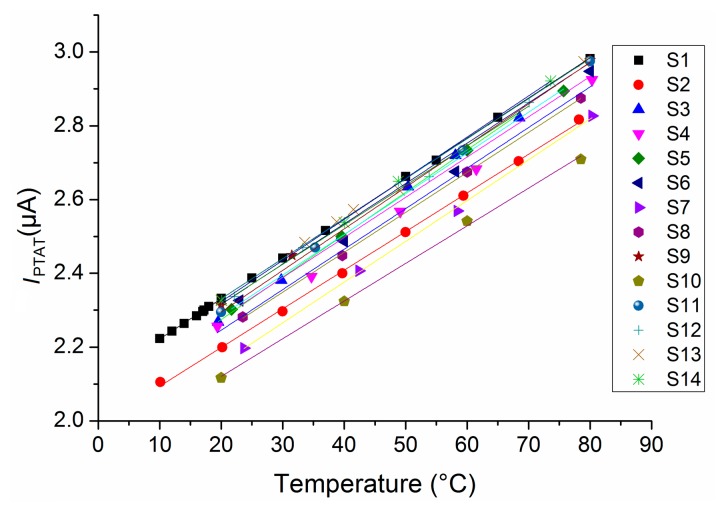
Measured PTAT currents *versus* temperature.

The measured PTAT current varies with temperature as expected (Figure 10):

(21) $$I_{\text{PTAT}}(\mu \text{A})=-0.011\cdot T({}^{\circ}\text{C})+2.05\mu \text{A}$$

where the 99% CI is (1.995, 2.098) μA for the intercept and (10.6, 11.2) nA/°C for the slope. This yields a normalized temperature coefficient of 0.011/2.05 = 5366 ppm/°C at 0 °C.

### 5.4. Measured Frequency Output (Version 2)

The measured conversion from current to frequency (TMOD) over the frequency range 20–70 °C produced oscillator output frequencies in the range of 90–108 kHz. Similarly, the measured conversion from capacitance to frequency (RHMOD) over the humidity range 20%–80% RH produced oscillator output frequencies in the range of 26.6–27.1 kHz. There was an anomaly in the intermediate range, and extensive post-layout simulations revealed that this was due to the shared bandgap reference (Figure 1) and can be corrected by providing isolated bandgap references.

**Figure 11.**
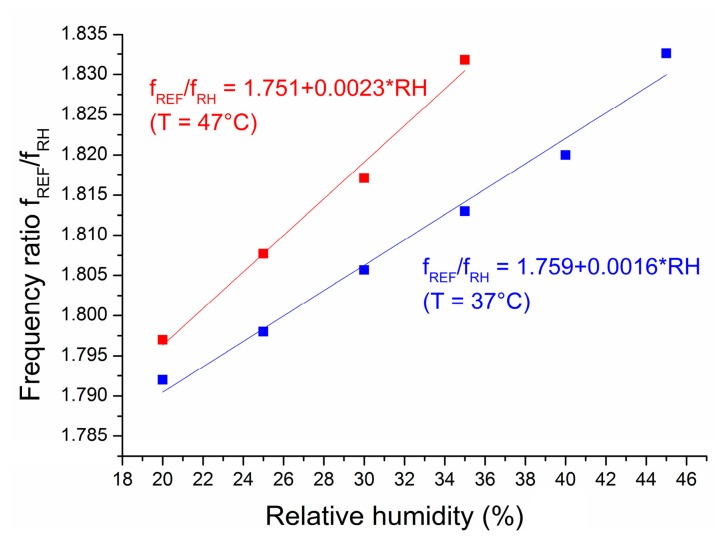
Measurement of frequency ratios in Version 2.

The behaviour of the measured oscillator frequency ratio between 20% RH and 45% RH for a representative chip from Version 2 is shown in Figure 11. The directly measured frequency ratio *f*_REF_/*f*_RH_ is shown. The ratio follows a linear trend with humidity, where the slopes are dependent on the temperature (0.0016/% RH at 37 °C and 0.0023/% RH at 47 °C). The normalized sensitivity at 37 °C and 0% RH is 909 ppm/% RH, where both intercepts nearly meet. The temperature error at 0% RH would be 1% RH per 2 °C, which is lower than what was expected from Equation (11). The reasons behind this temperature dependency are discussed in Section 6.

**Figure 12.**
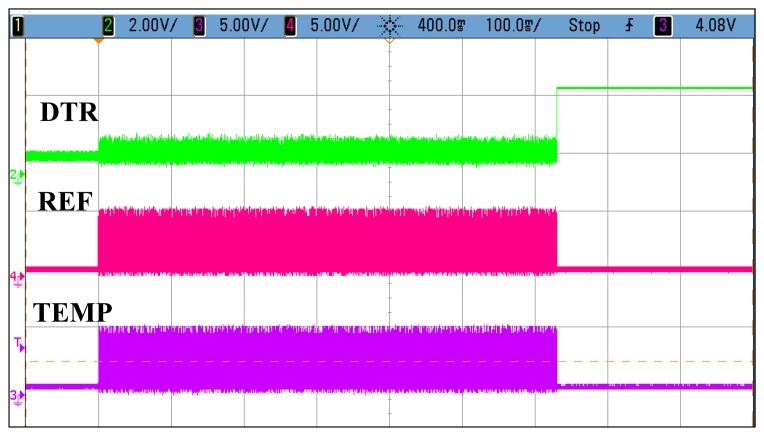
Oscillogram of the oscillator outputs. TEMP, temperature signal; REF, reference signal; DTR, data ready signal.

Figure 12 shows the oscillogram of the oscillator outputs in the temperature mode, where the reference oscillator correctly stops after 65,535 cycles. The DTR signal is set and is automatically reset after all of the data has been clocked out (not shown).

## 6. Discussion

### 6.1. Temperature Sensor

The temperature sensor was designed with two currents of opposing temperature coefficient. For the reference current, a linear function of temperature was measured with a negative temperature coefficient. The slope of the PTAT current was larger in measurements than in simulation (about 10%), which could be attributed to a 10% smaller resistor. This is within the tolerance of the polysilicon resistor (*R* in Figure 5b) as quoted in the process specifications. However, the ratio between CTAT and PTAT currents within the bandgap (BG) should only depend on the ratio between resistors (which can be accurately matched) and on the collector current ratio between *Q*_1_ and *Q*_2_ (Figure 5a). In theory, the latter should not depend on the process spread [13]. However, the collector current, *I_c_*, depends on both the emitter current and a significant base current (as the current gain *β_F_* is only 2.6 for the chosen process). Although the dependency of the current gain on the current is taken into account in the provided Gummel–Poon model, the recombination effect for the base-emitter diode presents a ‘knee’ in the base current *versus* voltage function and may not be accurately modelled [19]. Thus, the already small *β_F_* may significantly drop even further with smaller emitter-base voltage (*V*_EB_), so the collector current tends to be even smaller for each of the eight bipolar junction transistors (BJTs) in *Q*_2,_ reducing the *V*_EB_ of *Q*_2_ and, therefore, increasing *V*_BE1_–*V*_BE2_ and, consequently, the PTAT current. Failure to accurately model the drop in *β_F_* = f(*I*_c_) in the simulation could, therefore, explain the discrepancies between simulation and results. An appropriate correction of the collector current ratio would undoubtedly lead to better temperature stability of the reference voltage. Despite the suboptimal performance, the temperature coefficient of the bandgap voltage (316 ppm/°C) is still more than an order of magnitude lower than the temperature coefficient of the PTAT-to-reference current ratio. The oscillator frequency ratio is, therefore, mainly determined by the current, not the threshold voltages (*V*_1_ and *V*_2_).

The measured results confirmed the principle that the reference current can be designed to have a negative temperature coefficient with a low spread of slopes. As the slopes are related to the complementary temperature coefficient of the n-well resistors, it follows that their spread in the temperature coefficient must be low, as well. It was demonstrated that a compromise between linearity and sensitivity can be achieved by choosing the type of resistors *R*_1_–*R*_3_ (Figure 5a) on the basis of a desired temperature coefficient. Non-silicided P+ poly resistors, for example, can have a temperature coefficient as low as 40 ppm/°C, which would greatly improve the linearity at the cost of PTAT sensitivity.

### 6.2. Humidity Sensor

The measured capacitance is about 10 pF higher than was expected from simulation. The reason for this mismatch can be partly attributed to the parasitic capacitances. However, that may not be the only reason why the sensitivity of 0.0514%/% RH is lower than the previously reported value of 0.077%/% RH for a similar construction in a different CMOS process [15]. The lower sensitivity can be attributed to the sum of minimum track width and spacing, which was 3 μm + 2.5 μm = 5.5 μm (as defined by the design rules) in the 0.18-μm CMOS process. This is larger than the 5 μm in [15], yielding a 10% lower density of electrodes per area. Furthermore, the gap distance of 2.5 μm was lower than the computed optimum of 4 μm for a comparable electrode density [18].

Despite the reduced sensitivity of the sensing element, an LSB resolution of 1/124% RH is possible, due to the 16-bit counter resolution. Repeated measurements of 100 consecutive readout values at a humidity level of 20% RH showed a sample standard deviation of 65 counts; thus, a 3σ difference of 195 counts is detectable. A resolution of 2% RH is therefore achievable. It has been shown that for the entire batch, an absolute accuracy of 6% could be achieved for a span of 40% RH. For smaller sensor areas, the oscillator frequencies must increase, but this has the advantage of shorter conversion times. For Version 2 (300 μm × 300 μm sensor), the frequency was 1.8 MHz, and tens of MHz can be achieved with CMOS relaxation oscillators. A higher sensitivity (909 ppm/% RH at 0% RH) was found in that version, where the sensing capacitor was not accessible for capacitance measurement. This suggests that the sensitivity of the humidity measurement is not necessarily related to the total area of the sensor when using the same shape of its outline.

Measurements showed a temperature dependency (Figure 11) that is larger than expected (1% RH per 2°C instead of 1% RH per 18 °C), but comparable to [15], where the output frequency changed 0.14% per 8 °C or 0.0175%/°C. This corresponds to a normalized sensitivity of the frequency output of 0.045%/°C for a 1% change per 2.6 °C. The output frequency as a function of humidity in [15] was not only offset with higher temperatures, but its negative slope steepened, as well. This would mean that the sensitivity would increase with increasing temperature. The reasons for this may lie in the temperature dependence of the polyimide moisture absorption, which can be taken into consideration by a temperature-dependent correction factor for the relative capacitance change with RH [20]. Interpolation of the experimentally obtained values showed that the factor varied from 1.04 at 37 °C to 1.086 at 47 °C, corresponding to an increase of 4.6% change. Although this is much smaller than observed in the present paper or in [15], the temperature dependence of the humidity measurement is unlikely to originate from temperature-dependent oscillator bias currents. The argument being that: (i) the temperature coefficient of both reference currents should cancel each other out according to Equation (8); (ii) if the temperature coefficient of the reference currents were dominant, then their negative values would actually decrease the sensitivity with temperature; and (iii) the charge current in [15] was temperature independent.

### 6.3. Batch Calibration

The data for the entire batch suggests that the temperature measurement error due to the variation of the current slopes is small in comparison to the error due to the spread in the current intercept, that is, the offset. For example, the range of the 99% CI (confidence interval) of the intercept is 2.098 μA − 1.995 μA = 103 nA for the PTAT current and 2.95 μA − 2.81 μA = 140 nA for the reference current. However, in a temperature interval from 20 °C to 80 °C, the maximum error due to the slope deviation would be much lower, that is (11.2 – 10.6) nA/°C · 60 °C = 36 nA for the PTAT and (8.2 – 7.7) nA/°C · 60 °C = 30 nA for the reference current. It is, therefore, safe to say that for the short temperature range of interest, a one-point calibration technique would be adequate for the batch under consideration. This can be readily achieved by reading and storing the digital value at a defined temperature and subtracting this value in subsequent measurements.

The chip-to-chip variation in the RH measurement is dependent on the spread of the capacitive sensing element. The 99% CI intercept range is 0.19 pF, whilst the error due to slope deviation amounts to (0.0141 – 0.0125) pF/% · 100% = 0.16 pF for the entire measurement range. This suggests that the slope of the sensor should be calibrated in those applications that require a high degree of absolute accuracy, but the one-point calibration as described above would be sufficient to monitor excessive moisture ingress in micro-packages with a compromised hermetic seal. The calibration of the humidity sensor within the hermetically-sealed micro-package [14] could only be reliably performed once it is ensured that the internal humidity is close to zero and that the properties of the sensor are not affected by the bonding process.

## 7. Conclusions

A smart CMOS sensor with a combined readout for both temperature and humidity has been presented. Circuits were designed to meet the needs of low current consumption, high sensitivity, no post-processing and small size (see comparison in Table 2). These features were achieved using common circuitry for both temperature and RH measurements.

It was shown that the digital output of the sensors represents an indirect ratiometric measurement of frequencies. A linear response was achieved for humidity-related capacitance changes with a reasonable sensitivity. In this design, the implementation of a current source with a negative temperature coefficient achieved a much higher sensitivity than a conventional PTAT, at the expense of reduced linearity. The designer can find a compromise between sensitivity and linearity depending on the requirement of the application. In this application, the aim was to reliably detect small temperature changes in order to guarantee safe operation of an implantable chip and to set an alarm when the integrity of the hermetically-sealed micro-package is compromised by detecting any ingress of moisture.

Future work is necessary to determine the temperature-dependent sensitivity of the employed polyimide and how its sensitivity varies across an entire wafer. In addition, the high temperatures involved in the micro-packaging process [14] may have an irreversible effect on the short- and long-term vapour adsorption properties of the polyimide film, which should be also investigated.

## Figures and Tables

**Table 1.** Summary of performance.

**General**		

Technology	0.18 μm CMOS 5 M + THKMET	
*V*_DD_	+5 V	
*I*_DD_^a^	85 μA	
Area ^b^	0.2 mm^2^ (readout in both sensors)	

**Sensor Output**		

	Temperature	Humidity
Sensitivity ^c^	486/°C	124/% RH
Sensitivity error ^d^	< ±4.3%	< ±14.5%

**Temperature Sensor**		

Area	0.023 mm^2^ (part of readout circuit)	
Sensitivity	7753 ppm/°C@37°C	
Linearity	Compromise between linearity and sensitivity (see text); ideal for *TCI*_REF_ = 0.	

**Capacitive Humidity Sensor**		

	Version 1	Version 2
Area	1 mm^2^	0.09 mm^2^
Sensitivity ^e^	514 ppm/% RH	584 ppm/% RH

a Static quiescent current;

b readout only;

c as change in the counter output per measurement unit;

d maximum variation for the batch (N = 14) of Version 1;

e obtained by measurement of capacitive changes in Version 1 and by frequency ratio in Version 2.

**Table 2.** Comparison of CMOS temperature/humidity sensors.

**Reference**	**Type**	**Normalized RH Sensitivity (ppm/% RH)**	**Temperature Resolution (°C)**	**Current Consumption (μA)**	**Area (mm^2^)**	**CMOS Technology (μm)**
[12]	T	n/a	0.05	10	0.12	0.35
[13]	T	n/a	0.03	25	4.5	0.7
[21]	T	n/a	0.25	20	0.18	0.18
[15]	RH	770	n/a	111	4.8	0.6
[22]	RH	1440	n/a	1	0.7	0.15
This work	T/RH	584	7753 ppm/°C ^a^	85	0.29	0.18

a In this work, it is the sensitivity that is important.
